# Response of the Cutworm *Spodoptera litura* to Sesame Leaves or Crude Extracts in Diet

**DOI:** 10.1673/031.009.5201

**Published:** 2009-07-13

**Authors:** Henry Ofosuhene Sintim, Toru Tashiro, Naoki Motoyama

**Affiliations:** ^1^ Graduate School of Science & Technology: Chiba University, 1–33 Yayoi-cho, lnage-ku, Chiba, 263–8522, Japan; ^2^ Graduate School of Horticulture, Chiba University, 648 Matsudo, Matsudo-shi, Chiba, 271–8510, Japan

**Keywords:** varietal differences, crude extract, insect development, detoxification

## Abstract

The effects of extracts of sesame, *Sesamum indicum* L. (Liamiales: Pedaliaceae), and whole leaves of some selected cultivars of sesame were tested using a natural host *Spodoptera litura* (F.) (Lepidoptera: Noctuidae). Indices taken using the immature stages include; diet utilization, growth and development and induction of detoxification enzymes. The results indicate that *S. litura* generally selects its food amongst cultivars within 6 hours after food presentation. Growth and development of the insect is controlled also by plant acceptability and quality. Although all the cultivars tested significantly limit insect growth and development the variety 56S-radiatum did not allow a complete life cycle as pupation from first instar stage was 0%. Generally the crucial period for immature *S. litura* was the larval period, especially the first two instars where the weight of an insect fed on an experimental diet was three times lower than that of a control diet. The larval developmental period was greater than 40 days as compared to 17 days for insects fed a control diet. *S. litura* also had lowered efficiency in utilizing ingested food, from a low of 13% in a sesame cultivar to 45% in the control diet. The key detoxification enzyme was a glutathione s-transferase that was confirmed by a 6-fold increase between *S. litura* fed a plant cultivar vs. a control diet towards the substrate 1,2-dichloro-4-nitrobenzene. First and second instars of *S. litura* have a relatively reduced detoxification of enzymes in response to plant cultivar diets leading to low survival. A 3% v/w crude extract of the cultivars increased enzyme induction towards all the tested substrates.

## Introduction

The cutworm, *Spodoptera litura* Fabricius (Lepidoptera: Noctuidae), is a polyphagous insect that has about 150 host species (Rao et al. 1993). It is one of the most economically important insect pests in many countries including India, Japan, China, and other countries of Southeast Asia and has been recorded as a cosmetic pest of sesame in Japan (unpublished observations). *S. litura* is often used to evaluate antifeedants in plants (Wada et al. 1968).

Sesame, *Sesamum indicum* L. (Lamiales: Pedaliaceae), belongs to the Pedaliaceae which are not known as biopesticide candidates. The origin of sesame is said to be Sudan but it thrives with its numerous land races in most places in the southern hemisphere hence comparisons between authors working on sesame are often difficult due to normal biological variations relating to cultivar, seasonal, environmental and agronomic practices. Sesame is a plant whose seed and oil is said to contain abundant lignans primarily, sesamin (Kato et al. 1998; Fukuda et al. 1985) that are diphenolic compounds that have been studied in medical research and are thought to be biologically active (Ayres et al. 1990).

The physical attributes of sesame cultivars do not offer clues to ecological resistance as they are quite similar. We thought that the response of an insect could be used as a marker for differences amongst the plant cultivars. In situations where a plant has geographical plasticity, active factors could be concentrated in extracts using appropriate solvents (Gorinstein et al. 1994; Justesen 2000). The goal should be a clean extract with minimum interfering compounds such as chlorophyll (Shcherbakov et al. 2006). Plant allelochemicals could deleteriously affect the insect in diverse ways, such as through toxicity, and interference with the consumption and/or utilization of food (Lindroth 1991). The deleterious effects of certain purified phytochemicals or crude plant extracts on insects are manifested in several ways, including mortality (Hiremath et al. 1997), growth retardation (Breuer and Schmidt 1995), and feeding inhibition (Klepzig and Schlyter 1999). For example, Kunze et al. (1995) found that natural variation in amounts of farinosin and encecalin in *Encelia fannosa* was correlated with toxicity of leaves fed to *Spodoptera littoralis.*

Other reports also indicate that measures of insect development, behaviour and nutritional utilization lack the sensitivity to reveal subtle variations in host plants (Van Loon 1991), hence it is essential to develop a more sensitive biochemical marker, such as measuring the activity of a detoxification enzyme. Again, host plant induction of single enzyme systems has been observed in several insect species (Yu 1982; Kennedy et al. 1987), but few studies have investigated simultaneous changes in multiple enzyme systems (Lindroth 1989) and also within cultivars of a plant variety. This work thus presents the development-inhibiting activity of whole leaves or vegetative crude extracts of some selected sesame cultivars amongst a world collection using a natural pest, *Spodoptera litura* that avoided or utilized these cultivars in an earlier field selection trial. The goal is to clarify variability in enzyme induction within plant species and the multiple mechanisms that an insect employs to overcome plant diet burdens.

## Materials and Methods

### Source of plant material

Eight sesame cultivars that had been selected from amongst 54 in a field and bioassay tests due to their bio-active and insect aversion properties (data not shown) were used as the test botanicals. The selected cultivars were; 11Pusan (from Korea) 19Chinese-35 (from China), 24Nanbu-twasaki (from Japan), 29Bode-5 (from Myanmar), 45Laos (from Laos), 47Myanmar (from Myanmar), 53Sudan-534 (from Sudan), and 56S-radiatum (from Cameroon) had also been screened using diverse phytophagy insects for their ability to affect metabolic enzymes. The cultivar 45 Laos was included as a positive control as it supported larval development equally well as the artificial diet in preliminary experiments.

### Treatment of plants

Test plants were grown and harvested during the vegetative stage before flowering. The leaves and stems were air dried in a plant house at 40±5°C until there was no further change in weight. The moisture content of the plant parts was between 85–89% . This was then ground into powder using a blender and stored at -20°C until when needed. Leaves for bioassays were from potted plants in a plant house.

### Extraction processes

**Figure 1.**
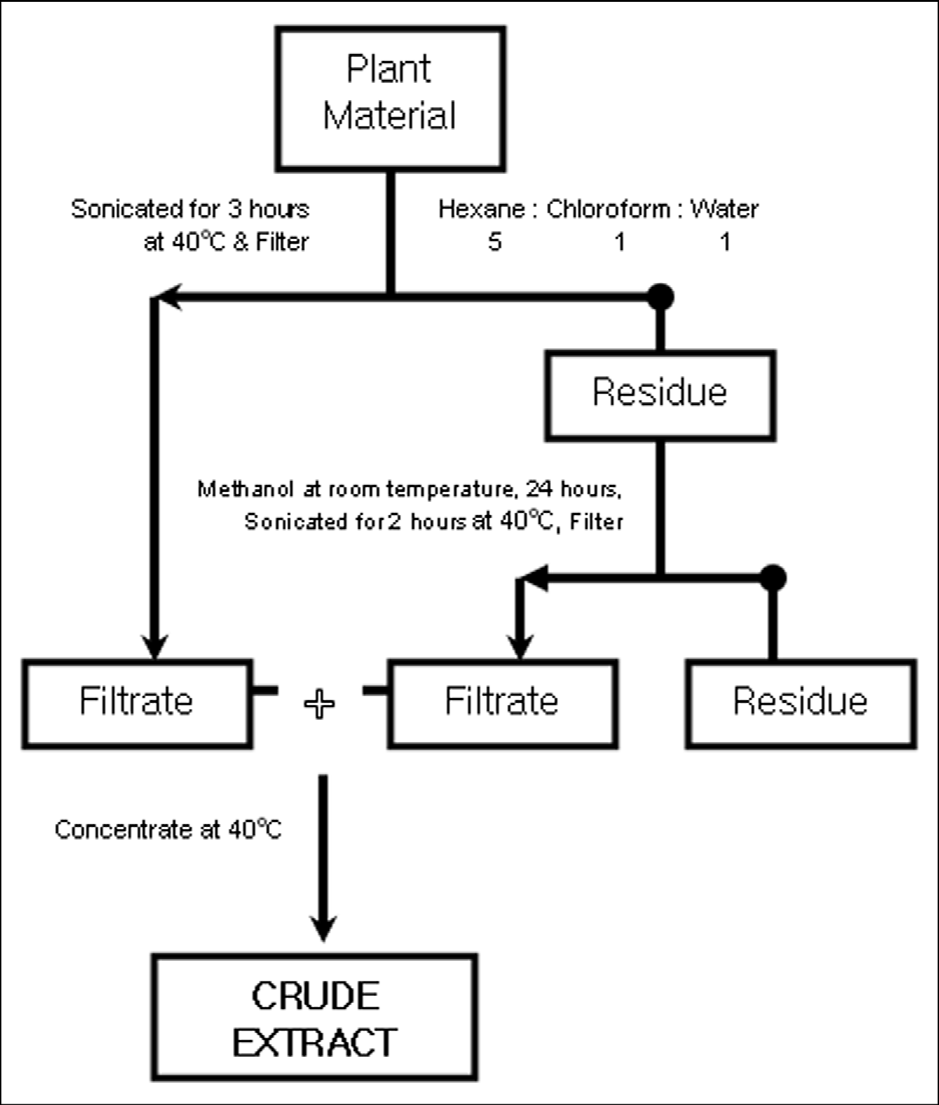
Schematic presentation of crude matter extraction from sesame leaves

10g of powdered material was extracted sequentially, first with 50 ml hexane, 10 ml chloroform and 10 ml water. It was sonicated for 3 hours at 40°C and filtered through an Advantec #5 filter paper (www.advantecmfs.com). The residue was then re-extracted with 50 ml methanol for 24 hours and then sonicated for 2 hours followed by filtration as before. The two filtrates were combined and filtered via suction and concentrated with a rotary evaporator (EYELA®) equipped with an aspirator (YAMATO®) at 40°C (Figure 1). The slurry extracts had yields between 3.3 and 14.9% volume by weight of initial dry matter. This formed the crude extract for the bioassays.

### Test insects

A susceptible strain of *S. litura* was obtained from Chiba Prefecture Agriculture Experiment Station, Toganeshi, Japan, and subsequently maintained on the artificial diet (Insecta LFS, Nihon-Nosan Kogyo Co. Japan) used at the Chiba Experiment Station at 23±3°C, 16L: 8D, light regime and 65% relative humidity. Experiments were done with respective instar larvae under the same environmental conditions starting after the second generation.

### Chemicals

Coomassie® brilliant blue R-250, fast blue B salt (FBBS), α-naphthyl acetate (α-NA), α-naphthol, sodium dodecyl sulfate (SDS), 1-chloro-2,4-dinitrobenzene (CDNB), 1 ,2-dichloro-4-nitrobenzene (DCNB), reduced glutathione (GSH), bovine serum albumin (BSA), dithiothreitol (DTT), sodium dithionite (Na_2_S_2_O_4_) and solvents were from Sigma Chemical (www.sigmaaldrich.com) and Wako (www.wako-chem.co.jp) at the highest purity available. Ethylenediaminetetraacetic acid (EDTA) was from Dojindo (www.dojindo.com), and carbon monoxide (CO) was purchased from GL Sciences (http://www.inertsil.com).

### Bioassay and biochemical procedures Choice tests

Larval preference amongst the cultivars was tested using a home-made perspex and paper arena. This was a cylindrical perspex chamber with a perforated lid. The cylinder was separated with a 0.5 mm thick cardboard into 9 discs and a circular central portion which housed the insects at time zero. Holes measuring 1.5 cm^2^ were made at the base connecting each of the 9 disc-shaped chambers (400 cm^2^) to the central area (500 cm^2^). Initially twenty-seven 3^rd^ instar larvae were put in the central chamber, with an option to select either a plant cultivar or a control diet placed in the connecting chambers. The test was repeated 15 times with new sets of larvae. Data were taken hourly for 20 hours.

### Insect growth and development

Using an initial number of 5 larvae per cup, larva and pupa weights and durations were measured. Whole leaves were fed to larvae starting with specific instars in 250 ml plastic cups and the survival after 5 days on the cultivars were determined. Insects were reared on an artificial diet to the needed instar before introducing them to the sesame cultivars or as control treatment. The initial number of larvae per treatment was 30. The experiment was repeated 6 times.

### Pupation

Pupation success was determined starting with 100 first instar larvae per cultivar. Pupation success was calculated as the number of the initial first instar larvae that pupated. The number of larvae per cup in all experiments was limited to five at the third instar stage to reduce or avoid cannibalism.

### Food consumption

Consumption of the sesame cultivars was determined using third instar larvae. Five larvae were fed ad libitum with each sesame cultivar. To avoid inconsistencies due to moisture loss, the weight of food consumed was determined by tracing the area consumed on filter paper as a function of the initial area before feeding. Food intake was taken as the dry weight of initial leaf minus the leftover. Due to reports (Candy and Baker 2002) disputing the fresh weight of food reported by Walbuaer (1968), the dry weight of the cultivars was determined by their characteristic moisture contents, which was between 85 and 89%. The fresh weight of insects was corrected to dry weight by comparing with groups of insects reared under the same conditions as test insects and then air dried at 100°C (Abdel Rahman 2001). The live body weight of each larva was recorded daily for 5 consecutive days. Frass was collected daily and weighed. Efficiency of conversion of ingested food (% ECI) was calculated as (dry weight gain/dry food consumed) × 100. The experiment was repeated 5 or 6 times.

### Detoxification enzyme induction

Enzyme induction experiments were conducted starting with various stages of the insect using either whole leaves or extracts in artificial diet. In experiments that were started with either second or third instars, the earlier larval stages were fed an artificial diet. First instars after 5 days are almost 2^nd^ instars and hitherto are referred to as 1^st^ to 2^nd^ instars, whilst 2^nd^ instars could reach 3^rd^ instars at the end of the feeding tests and are referred to as 2^nd^ to 3^rd^ instars. Tests that were started with 3^rd^ instar larvae are referred to as post 3^rd^ instar larvae.

Starting each with either first or second instar larvae, they were fed with whole leaves of the sesame cultivars at ad libitum for five days and whole bodies of these early instars were used for tests.

Third instars were fed with crude extracts from the cultivars at 3% v/w inclusion in an artificial diet or fed with whole leaves for 5 days. The inclusion rate was based on preliminary trials. Mortality was observed at 3% v/w in two of the cultivars. The 3% extract inclusion rate represents approximately forty times the average daily wet leaf intake of 500 mg. The above insects after feeding formed the tissues used for enzyme induction assays.

The activity of non-specific carboxyl esterase (CarE), glutathione s-transferase (GST) and mixed function oxidases (MFO) was estimated in the experimental insects. Cytosolic fractions of whole bodies were used in comparing 1^st^ to 2^nd^ or 2^nd^ to 3^rd^ instar larva fed with the same cultivar and also amongst the cultivars. Microsomal fractions of midgut homogenates were used in comparing post 3^rd^ instar larvae that had been fed with the cultivars either as concentrated crude extract in diet or as whole leaves for five days.

### Tissue Preparation

All steps were carried out at 4°C on ice. Insect tissues were homogenized in ice-cold 0.1M sodium phosphate buffer, pH 7.2 using a Teflon homogenizer. In the homogenizing buffer for whole insect homogenates, 1mM EDTA and 1mM DTT was added. The homogenate was centrifuged at 12,000 × g for 20 min at 4°C (Hitachi 20PR-52) and the fatty layer was removed using glass wool and was used fresh or stored at -20°C prior to enzymatic analysis of first and second instars. Microsomes of post 3^rd^ instar larvae were isolated from the larva midguts by differential centrifugation according to Crankshaw et al. (1979). Briefly, microsomal fractions were prepared from the 12,000xg supernatants by further centrifugation at 120,000×g for 60 min (Hitachi CP B5β). The resulting fractions were used fresh. Protein concentrations were determined by the method of Bradford (1976), using bovine serum albumin as standard.

### Enzyme Activities

Whole insects or midguts were homogenized in 4–6 volumes of 0.1M phosphate buffer, pH 7.0 in micro centrifuge tubes. The reaction mixture consisted of 150 µl crude homogenate in 1.35 ml 0.27mM α-naphthyl acetate, which was incubated for 20 minutes at 30° C. The reaction was terminated and dyed by adding 500 µl of 1% fast blue B Salt and 5% sodium dodecylsulphate solution in a 2:5 ratio v/v. After the fast blue B salt reacted with l -naphthol which resulted from the hydrolysis of αnaphthyl acetate, the developed colour was measured with a Shimadzu UV-recording (160A UV) spectrophotometer at 600 nm (www.shimadzu.com). One unit of enzyme activity was defined as the amount of enzyme that produced 1 µmol of α-naphthol per min at 3O°C. The extinction coefficient was determined as the slope of the concentrations of α-naphthol (0 to 100 µmol/ml). Nonenzymatic hydrolysis was converted by substituting the enzyme with phosphate buffer as the reference.

The crude homogenate was diluted as appropriate and GST activity towards the general CDNB and specific DCNB substrates were assayed according to Habig et al. (1974). The reaction mixture consisted of 1ml of 1mM substrate, 1ml of 1mM GSH, 1ml buffer and 0.1ml crude homogenate in a total digest of 3.1ml. Enzyme kinetics was recorded over 5 minutes at 30 second intervals. The formation of S-(DNP)GS was continuously monitored at 340 or 344 nm. One unit of enzyme activity was defined as an initial rate of 1 µmol S-(DNP)GS formed per minute using an extinction coefficient of 9.6 or 10 /mM/cm for the conjugate of CDNB or DCNB respectively and activity was expressed in unit mol/ min/mg protein. All measurements were adjusted for by the nonenzymatic conjugation of either substrate in the reference samples (Coleman 1997).

Quantitative determination of cytochrome P450 in microsomes from 120,000 × g pellets of post third instar larvae in pH 7.4 re-suspension 0.1M phosphate buffer was made according to the method described by Omura et al (1964) with a Shimadzu UV-instrument using an extinction coefficient of 91/mM/cm. Following treatment with sodium dithionite, the microsomal suspension was carefully bubbled with CO in a fume chamber. The difference in spectra absorbance (400–490 nm) between the sodium dithionite-reduced enzyme and a CO complex was recorded and specific content of P_450_ was expressed as unit moles/mg protein.

### Data Analysis

Larva survival (number alive at the end of a test) of either first or second instars are expressed as a percent of the starting number of insects. Percent pupation from the first instar stage to pupation is the proportion of successful pupa in reference to starting larvae numbers. Weights of insect, food consumed and frass are expressed as means ± standard deviation. Efficiency of conversion of ingested food (ECI) is expressed as weight gained/food consumed. The Friedman test (χ^2^) for the homogeneity of ratios was used to test the significance of diet selection between the control and cultivars with the assumption that the initial 27 larvae select the 9 treatments equally. Variances in insect development indicators and metabolic enzyme induction were determined by a one-way non-parametric test of multiple comparisons among the treatments, including the control. This tested the null hypothesis of no differences amongst the variances of the treatments using XLSTAT 2007. Means were separated using multivariate comparison procedures. A student's t-test was used to compare differences among paired means of the effects of the leaves and extracts of the same cultivars or different instars fed on the same variety.

## Results

The choice test showed that a significant (P ≤ 0.01) and increasing proportion of the larvae preferred artificial diet over the 20 hours of the test (Table 1) which may simply reflect a preference for the diet on which they had been reared. By 20 hours there were few significant differences in attraction to the different cultivar leaves. Attraction to the cultivar 45Laos, the positive control, was not significantly different from the artificial diet (χ^2^ (8) 63.98 P = 0.0014). Attraction to variety 56S radiatum was lower than the other cultivars, but not significantly different except that by the 6th hour that its attraction was significantly different from the cultivar 53Sudan-534 (Table 1).

The assay of toxicity depended on the comparison between the growth efficiency of larvae fed on sesame leaves and the growth efficiency of larvae fed on the control artificial diet. The ability of third instars to use sesame leaves was measured in the form of efficiency of conversion of ingested food (% ECI), which showed significant differences (P ≤ 0.05) between the control diet and the leaves (Figure 3). The difference in ECI, calculated over a period of 5 days, between 3^rd^ instar larvae supplied with sesame leaves and those kept on the artificial diet was high. Diet intake and frass output were comparable to the control but due to the fact that the leaves have higher moisture contents of between 85 and 89%, the insects tend to consume a lower amount of dry matter. The variety 56S-radiatum consistently had a low intake and low frass output as well as the lowest ECI (13%) as compared to 46% for the control diet (Figure 3). The most inconsistent cultivar, 24Nanbu-twasaki which had the highest leaf intake (560 mg/day) and a low frass output, yet had an ECI of only 16% indicating that it was the poorest diet and contributed the least to insect body mass. These results indicate that the leaves present diverse diet qualities as well as antifeedants. For example, larvae on 56S-radiatum had a feeding pattern where they selected the mid rib and refused to consume the leaf lamina (data not shown).

To obtain a streamlined diet intake amongst the cultivars the moisture of the leaves was removed by air drying them over 14–18 days. The moisture content of the cultivars was in the range of 85–89 %. When the dry matter was further extracted with stepwise organic solvents (Figure 1) the yields were variable with a range of 4–15% (v/w).

Data taken for developmental indices were larval weight and duration and pupa weight and duration. The results presented in Table 1 show that the sesame leaves were either antifeedants or reduced insect development. The sesame leaves also had a strong effect on the survival. For example, the mortality of larvae that fed on leaves of variety 56 S-radiatum reached 100% by the 11th day. Body weight of larvae feeding on sesame leaves was significantly lower than those feeding on the artificial diet (F-test 8, 114.19 P ≤ 0.05) after 11 days and at the fifth instar stage (Table 1). The larval development period was significantly (F-test 7, 51.64 P ≤ 0.05) prolonged for larvae feeding on sesame leaves as compared to the artificial diet. Whilst larvae feeding on the artificial diet had larval periods of 17 days, those feeding on sesame leaves were greater than 40 days. The shortest larval period (17.5 ± 1.2 day) was observed among the larvae fed on artificial diet while the longest period (67.7 ± 5.3 day) was that of larvae fed on 45 Laos (Table 1). Pupa weights were also significantly lower (F-test 7, 43.38 P ≤ 0.05) than that of the control treatment (Table 1). The low weight of the pupa amongst the cultivars did not however contribute significantly (P >0.05) to pupa duration.

**Table 1.**
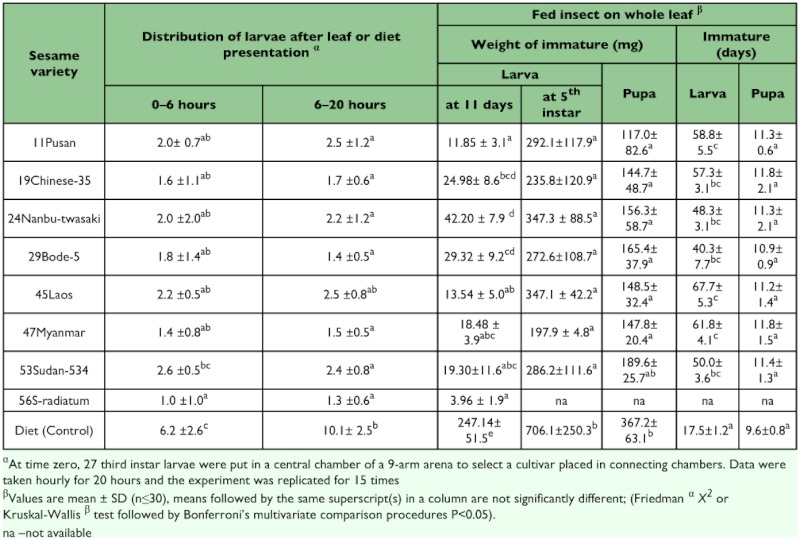
Feeding and development of *Spodoptera litura* immature stages on diet or leaves of eight sesame varieties

**Figure 2.**
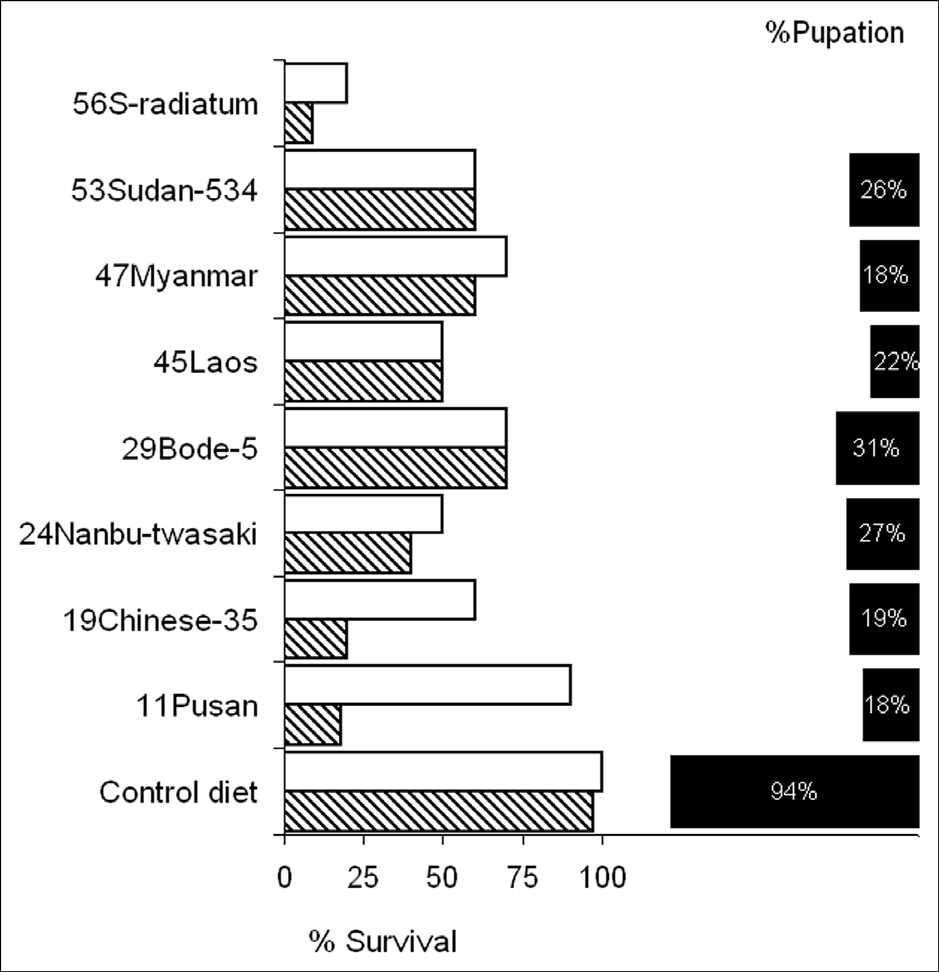
Survival beyond five days of the first 3 instars and pupation success from the first instar of *Spodoptera litura* fed with sesame leaves or a control diet. Hatched bar = First instar, open bar = Second Instar, solid bar = % Pupation. Second and third instars were initially reared on the control diet (n=30). % Pupation is the ratio of larvae that successfully developed from first instar to pupa with an initial number of 100 larvae.

Emerged adults from insects feeding on sesame leaves were relatively smaller (≤ 120 mg) in contrast to live body weights greater than 320 mg in insects fed with the artificial diet. The critical index in pupa growth was eclosion as pupation rate was significantly higher (P <0.01) in the control diet (94%) as compared to 0–31 % amongst the leaves (Figure 2). Feeding on sesame plants reduced the duration of the moult cycle, and thus, growth. The survival of larva on the leaf diets at the various instar stages shows that the critical stages are the first and second instars. Once an insect gets to the third instar, its survival to pupation was high (Figure 2).

The induction of detoxification enzymes after feeding on either whole leaves or crude extracts mixed with an artificial diet gave respective significant differences against a control diet and also amongst the cultivars. General esterase activity showed a slightly different pattern than glutathione s-transferase (GST) activity between instars. There was however an increased enzyme induction from leaf to extract diets towards all substrates when larvae were fed on either a leaf diet or a concentrated extract with the diet as media.

Generally between the first to third instars, whilst general esterase induction significantly decreased (t-test (52), 11.17 P <0.05) the induction of GST increased significantly towards CDNB (t-test (52), 9.04 P <0.05) but not when DCNB was substrate (t-test (52), 1.36P = 0.18) (Table 2). The reduced, general esterase activity from first to third instars may be a short-term phenomenon related to reduced feeding on a novel host or to inherently poorer nutritional quality of the leaves compared with the inherited ability to feed on an artificial diet. When the change of enzyme induction was tested (t-test) in individual cultivars, it could be seen that the treatments including the control had decreased esterase induction after the first instar (Figure 4). The decrease in two of the cultivars, 47Myanmar and 29Bode-5, were however not significant (t-test (4) P=0.14 and 0.08 respectively). This pattern was not the case for the GST substrates (Figure 4). The cultivar 47Myanmar, for example, had a 6-fold significant increase towards both GST substrates when post 1st and post 2^nd^ instars were compared.

Enzyme induction amongst the cultivars towards the substrates was variable in the first to second instars (Table 2). Esterase induction was not significantly different between the control and the leaves, but two cultivars, 11Pusan and 19Chinese, have induction levels that are significantly higher (F-test 8, 21.59 P <0.05) than the variety 56S-radiatum. In the second to third instar larvae, general esterase induction was significant amongst only three of the cultivars. The induction of GST amongst the cultivars was more pronounced towards CDNB, where 45Laos, the positive control induced significantly lower (F-test 8, 23.02 P = 0.01) than 47Maynmar and 56S-radiatum in 2^nd^ to 3^rd^ instars. The control diet had a lowered GST induction compared to the cultivars except for the cultivar 45Laos. In 2^nd^ to 3^rd^ instars for example there was a 6-fold significant increase (F-test 8, 23.30 P=0.01) between 56S-radiatum and the control artificial diet (Table 2).

The comparison between midgut enzyme induction when post third instar larvae were fed with whole leaves or extracts (Table 3) clearly indicates a significant increase (P < 0.01) in both general esterase and GST induction when fed with the extracts. The 3% v/w extract represent 40 times the normal consumption of allelochemicals from leaves. Enzyme induction changes between whole leaf diets and extracts in artificial diet for individual cultivars are presented in Figure 4. All cultivar extracts had a significant increase (t-test (4), P <0.05) towards α-NA when fed as fresh leaves. For the GST substrates, CDNB has a general induction increment from leaf to extract. The substrate DCNB had a significantly (t-test (4), 4.55 P <0.01) reduced induction ratio for 56S-radiatum between leaf and extract diets (Figure 4), indicating that the leaf diet induced a higher GST enzyme towards DCNB for this variety.

**Figure 3.**
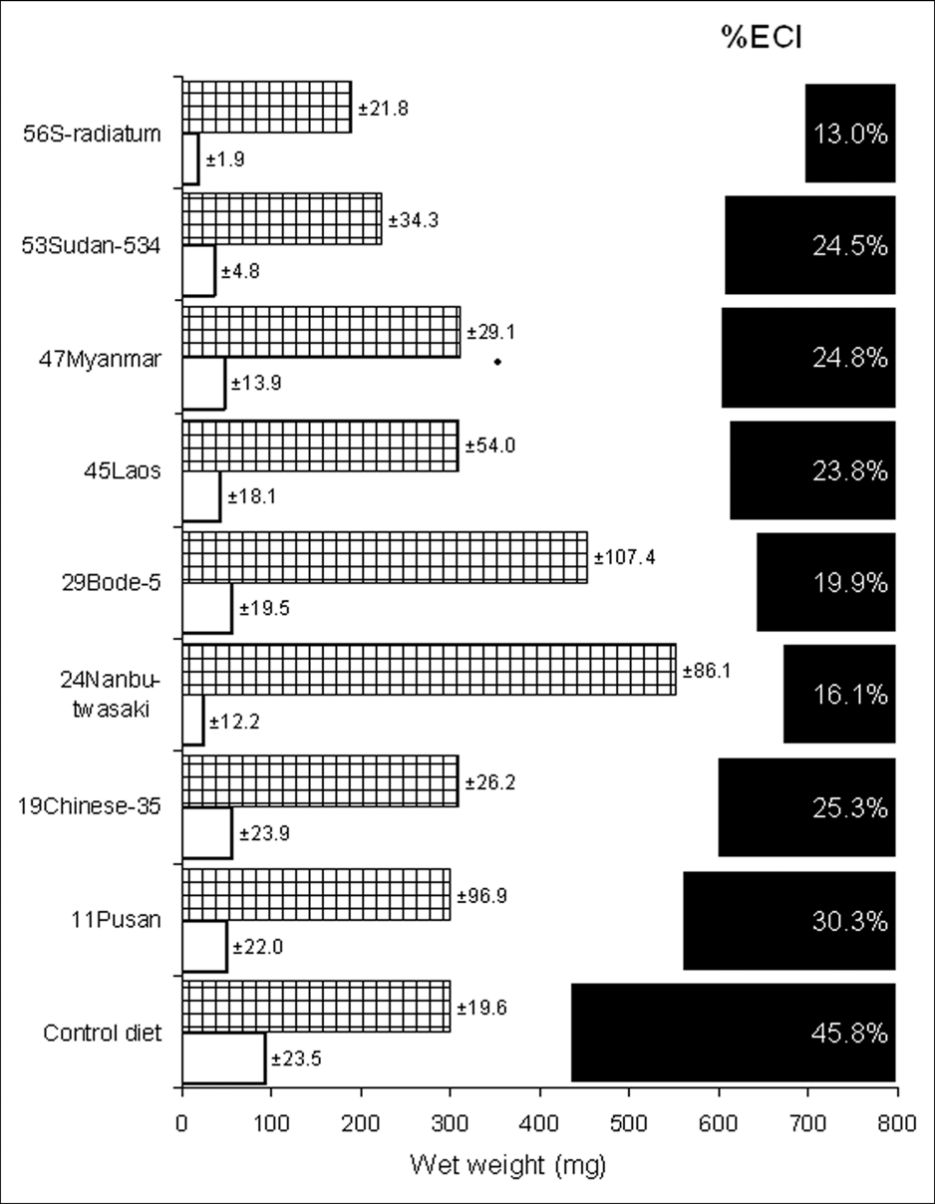
Food utilization per day of 3rd instar larvae of *Spodoptera litura* fed with sesame leaves for 5 days. Hatched bar = Weight of frass output, open bar = Weight of food intake, solid bar = % ECI. Bars are with means ± SD (n=30) ECI efficiency of conversion of ingested food by 3rd instars for 5 days: (weight gain/food consumed) × 100, (n=25).

Although mixed function oxidases are implicated in most detoxification mechanisms in response to plant diets, only 47Myanmar was consistent in inducing total monooxygenases in both leaf and extract diets (Table 3). The P450 content induced by 47Myanmar diets was about 3-fold higher in the leaf diet than the concentrated extract diet. Three other cultivars also induced total P450s.

## Discussion

Sesame is recognized primarily because of its health promoting properties. Most of the biological effects of sesame are also linked to either its seed or oil content. Laurentin et al. (2003) showed that compounds such as phenols, flavanoids, saponines and acids in sesame leaves were the key factors in whitefly repellency. Cattle fed excessively on sesame meal developed eczema associated with loss of hair and itching (Steyn 1934; Watt and Breyer-Brandwijk 1962). It has been reported that cattle and deer avoid sesame plants when grazing fields (Oplinger et. al. 1990). Insects survive on toxic plants because they specialize on certain plant families, species, or even a plant part and detoxify the toxic compounds present (Berenbaum 2002).

Even though insects are able to grow and develop on a variety of host plant species, their performance may be impaired on some species by reduced growth and larval survival, abnormal pupal development, delayed adult emergence, decrease in adult weight and, a reduced number of generations (Tikkanen et. al. 2000).

**Table 2.**
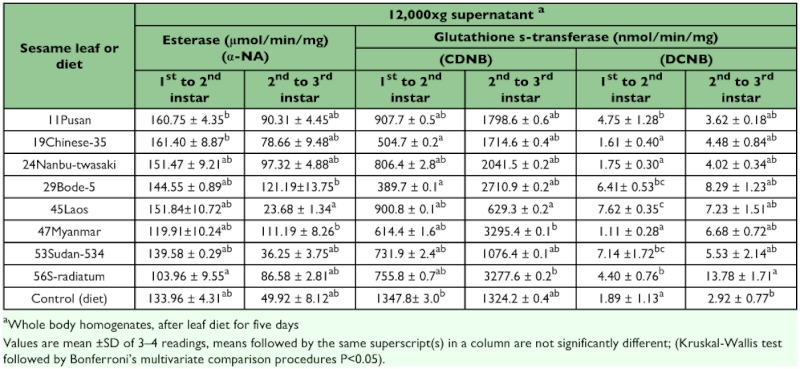
Enzyme induction of the first to third instars of *Spodoptera litura* after leaf diet

The leaves of the sesame cultivars were found to have conspicuous antifeedant effects on the larvae of *S. litura.* Although the feeding behaviour of larvae was not directly observed the experiments showed that the larvae selected its food, suggesting that the reduced survival was a consequence of either rejection without substantial ingestion or the presence of toxic compounds. Antifeedant substances have been classified by Chapman, (1974) and Schoonhoven, (1982) into repellents (which repel an insect without making contact with the material), suppressants (which suppress biting activity after contact) or deterrents (which deter an insect from further feeding after ingestion of the material). Using these definitions the sesame leaves had both suppressant and deterrent properties.

Since the appearance of Waldbauer's (1968) and Gordon's (1961) definitive treatments of quantitative nutrition of insects, numerous studies have appeared reporting consumption and utilization values in many kinds of insects and several kinds of food. It is evident that food consumption and efficiency was reduced with regard to the sesame cultivars for which the most plausible explanation could be attributed to the existence of antifeedants or a low food quality. We used %ECI based on Lindroth (1993) because it represents efficiencies of both digestion and how well digested food is converted to biomass. Slansky and Scriber (1982) found that the utilization efficiencies (ECI) for 11 species of predaceous insects were between 4 and 75%, hence the strain of *S. litura* used in this experiment compares favorably having an ECI value of 45.8 % when fed with the control artificial diet. Fraenkel (1981) argued that under the best conditions, a growing insect could convert a maximum of 2/3 of its ingested food to body materials with 1/3 going to metabolic processes. He said, further, that this seems to be the limit of efficiency in insects in general which Calow (1977) and Schroeder (1981) also confirmed. An ECI below 33% as was obtained for all the sesame cultivars tested may indicate the presence of limiting factors that in this case should be attributed to inherent properties of the cultivars rather than the insect. It seems reasonable that the high conversion efficiency with the artificial diet is associated with the high feeding volume. The variation in conversion efficiency amongst the cultivars was related both to variable food quality and pre-ingestion acceptance.

The early instar stages of insects are particularly sensitive to overall physiological and biochemical functions. Early instars either employ inherent capabilities or perish. This early sensitivity has been observed by Scriber (1981) in feeding experiments with *Spodoptea eridania* larvae. In the present investigation, the variety 56S-radiatum significantly decreased or halted the feeding activity and larva development. Although there are reports of acute toxicity of plant allelochemicals to adult beetles (Coyne and Lott 1976), the highest toxicity resulting from the 3%v/w crude extract in this investigation was due to two of the sesame cultivars at the preliminary stages during the determination of the inclusion rate. Feeding is obviously necessary for the stimulation of enzyme activities (Sibley 1981; Broadway and Duffey 1988) hence there was also a need to compare the effects of the leaves and extract of the leaves mixed with the artificial diet that has a higher intake. Using the artificial diet also enabled us to increase the amount of extract consumed.

**Figure 4.**
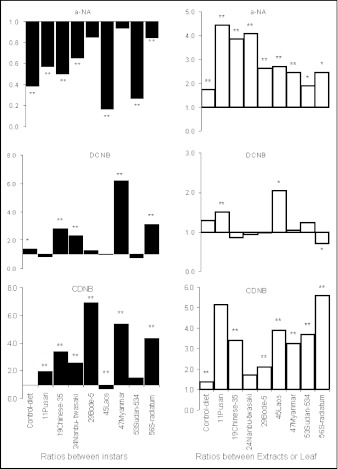
Change in detoxification enzyme induction between instars and also between whole leaf diets and plant extract diets towards various substrates. The bars represent the ratios of enzyme induction towards the respective substrates with the base line for no change at l on the y-axis: This is basically the row comparisons of Tables 2 and 3. Solid bars = 2^nd^ to 3^rd^ instar induction / 1st to 2nd instar induction, open bars = 3% v/w Extract induction / Whole leaf induction. Significant differences between induction ratios are denoted by *(P<0.05) and ** (P< 0.01) t-test.

The biotransformation of plant toxins is one of the major weapons that insects have evolved in their coevolutionary arms race with plants (Berenbaum 2002). There is a high variation in the ability of insects to metabolize the multiple compounds they encounter in a plant (Berenbaum and Zangerl 1998). Insects that are resistant to plant toxins usually combine several resistance traits for example, behavioural and metabolic (Després et al. 2007). Carpenter et al. (2002) suggested that the poor performance of a diamondback moth strain on the host plant was due to a reduced ability to detoxify the plant foliage allelochemical(s), which had a deleterious effect on the conversion of absorbed food to biomass. The validity of using artificial diets in antifeedant assays (Jermy 1990) and other claims that leaf disc bioassays may be more reliable because the quality of the plant's surface plays a crucial role in determining the acceptance or avoidance (Eigenbrode and Espelie 1995) should be considered cautiously. In situations where effects after ingestion are needed, the mode of presentation should be that which the insect accepts thus ensuring an even distribution of the toxicant. Assays eliminating the influence of starvation (Blau *et al*. 1978) due to leaf surface properties are a better option to measure the after-effects such as detoxification.

**Table 3.**
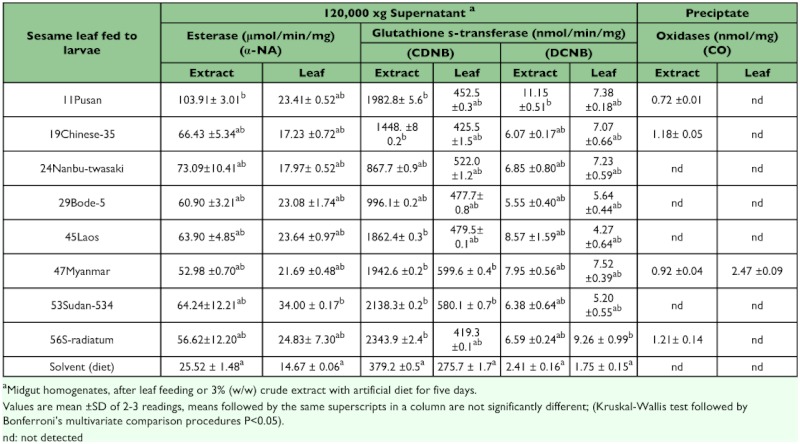
Enzyme induction of post third instar *Spodoptera litura* fed with either whole leaf or with plant extract in diet

Generalists such as *S. litura* may exhibit greater inducibility and range of responsiveness of enzymatic detoxication systems than specialists (Lindroth 1991). Induction of mid-gut esterases due to plant allelochemicals have already been reported in *S. litura* (Mukherjee 2003). The data presented here indicate that midgut enzyme activities are strongly influenced by the sesame cultivars consumed by *S. litura* larvae. Reports by Lindroth (1989) indicated very moderate induction of soluble esterases and substantial induction of microsomal esterases in response to food plant species. In our experiment, there were high levels of esterases in the soluble fractions and especially in the midgut homogenates.

The enzymes involved in detoxification pathways act on a broad array of substrates, including both naturally occurring plant allelochemicals and artificial pesticides (Gordon 1961). The physiological responses of herbivores to host plants leads to enhanced metabolic mechanisms in the detoxification of allelochemicals in their diets. Yu et al. (1979) reported increases of as much as 45-fold in cytochrome P450 enzyme activity in the variegated cutworm as a result of feeding on peppermint leaves. Different resistance mechanisms of insects maintained on specific plants have been related to different levels of metabolizing enzymes, presumably induced by the plants (Yu 1983). In addition to the antifeedant activity of sesame leaves, the variety 56Sradiatum exhibited chronic toxicity against *S. litura* larvae that was demonstrated by the death of all larvae reared on the leaves of this variety. Other examples were the low survival rates observed among larvae fed with the other cultivars, and the lower utilization efficiency (%ECI) of larvae fed on sesame compared with those fed on the control artificial diet. More food was being metabolized for energy and less was being converted to body mass. Added stress on the enzyme expression system to synthesize new and higher amounts of detoxification enzymes could be the possible reasons for the arrested growth and mortality. A decreased nutritional index is seen as a consequence of post-feeding toxic effects in *S. litura* (Wheeler and Isman 2000). Generally a growth regulator such as juvenile hormone could be assumed to be the cause of the long larva periods and the high non-specific esterase induction. However until a phytojuvenoid is confirmed in these sesame leaves, it is a conjecture at this stage. There have been instances reported by Howard et al. (1986) where peaks of α-Na activity and juvenile hormone esterase activity occurred out of phase in the migrant insect *Anticarsia gemmatalis.* Since disruption of normal growth in insects using juvenile hormones have relatively low adverse effects (Novak 1975), a discovery of a JH-compound from a food crop could be a break through in public health. It is known that GSTs are increasingly activated as larvae develop (Ahmad et al. 1986). This might explain the high changes in GST activities for the sesame cultivars in the older larvae except for 56S-radiadum that possesses the highest growth inhibition. Although *S. litura* was highly variable in its response to the diets, the evidence points to the fact that, with increased intake of a purified extract, the specific detoxification enzymes that the larvae produce after feeding, especially on 47Myanmar and 56S-radiatum, increased.
